# Telemedicine: A New Frontier in Clinical Practice

**DOI:** 10.12669/pjms.37.2.3592

**Published:** 2021

**Authors:** Munazza Asad, Nooresahar, Saniya R. Sabzwari

**Affiliations:** 1 Dr. Munazza Asad, Resident Year 3, Dept. of Family Medicine, Aga Khan University Hospital, Karachi, Pakistan; 2 Dr. Noor-e-Sahar, Resident Year 4, Dept. of Family Medicine, Aga Khan University Hospital, Karachi, Pakistan; 3 Dr. Saniya Raghib Sabzwari, Associate Professor, Dept. of Family Medicine, Aga Khan University Hospital, Karachi, Pakistan

**Keywords:** COVID-19, Telemedicine, Teleconsultations, Telehealth, Tele Guidelines

## Abstract

The COVID-19 pandemic has highlighted the important role of telemedicine as a tool for safe healthcare delivery across the world. While its use was more common in the developed world, the developing world has also adopted this strategy. It is important to develop a clear process and contextual guidance for effective use of this strategy for better patient-doctor interaction and its role in teaching/learning of trainees.

## BACKGROUND

Telemedicine is provision of healthcare using technology from a distance,^1^ via a telecommunications infrastructure.^2^ Telehealth is a broader term that covers all components and activities of healthcare and the healthcare system that are conducted through telecommunication technology. In the last few years advances in technology made telemedicine more common with approximately 100,000 telemedicine consults/month in developed nations.^3^ Telemedicine has also been used in developing countries to connect with tertiary hospitals.^4^

In Pakistan doctors started using email and telegram to consult their colleagues within and outside the country.^5^ Recent advancements in communication technology initiated telemedicine projects here as well, e.g. Sehat Kahani, a network of E-Health Clinics providing virtual consultations.

Telemedicine has emerged as a frontline strategy for safe healthcare delivery during the COVID-19 pandemic and is being promoted by several healthcare organizations across the world for cost- effective, safe and strategic medical care with clear guidelines for use.^6^-^8^

The Joint Commission for Healthcare accreditation has recently released an advisory on telemedicine, encouraging its use to improve patient access, maintain social distancing and reduce use of personal protective equipment. Healthcare providers do need to remain cognizant of certain challenges like non-uniform access to devices or internet and lack of familiarity with technology use.^9^

For an effective tele-medicine practice, a clear procedure for patient- doctor interaction becomes important as local and contextual guidelines are not available. This article suggests instructions and guidance for an effective tele-consultation.

### General Guidance

Whenever possible video technology should be used for visualization of a patient’s appearance and acuity of condition. Confidentiality takes on different meaning in a tele-consultation; a provider should ensure the safety of surroundings at both ends (patient and physician) and safe connections to ensure security of shared information.^10^

The following information is divided into sections keeping the process of consultation in mind.

### Pre-consultation instructions

- When using a laptop place it on a desk and sit on a chair in front of it.
- If using a phone place, it against an object two feet away from you and tilted at an angle of 120 and sit on a chair if possible.
- Have a pen/paper to note instructions.
- Have medicine list or medicine box and all investigations at hand.

### Initial part of consultation:

- To ensure a smooth interruption-free consultation we recommend that other phones be kept silent, the doctor and patient’s door be shut for privacy and noise reduction.
- If connectivity is poor, it may be feasible to do a video introduction and then take a history without video, returning back to video for physical examination or instruction sharing.
- Lighting in both the rooms (doctors/patient) should be adequate. There should be a light above or on the side of the patient. Lights from behind create halos and glare and should be avoided.^11^

- Make sure that entire family is not in the same room; preferably only the patient and one attendant (if needed).
- Ensure that you can be heard and/or seen clearly
- Meet and greet (ensure introduction of any staff/ trainee present with you)
- Confirm identity: Name/Medical record number
- Ensure that one person (doctor/patient/attendant) speaks at a time.
- If you document notes during consultation inform the patient that eye contact maybe interrupted.

### During consultation

History in a tele-consult follows the format of a face to face consult.^12^ Some components of physical examination can be attempted during a tele-consultation either by instructing the patient or with the help of an attendant. Table-I suggests possible physical examination options.

**Table-I:** Physical examination options in a tele-consultation.

*Examination*	
Vitals	• Temperature: Thermometer (if relevant and available)
	• Heart rate can be gauged by placing hand on patient’s chest and counted for a minute.
	• Respiratory rate can be measured by asking a patient to lie supine and placing a piece of paper on patient’s abdomen to count the number of times the paper moves up and down in 30 or 60 seconds.
General physical	• Cyanosis, gross edema and large goiters maybe visulaized.
***Systemic Examination***	
Cardiovascular	• Orthopnea may be seen when patient is supine
Respiratory	• Inability to complete a sentence, visualization of accessory muscle use or an audible wheeze may help determine respiratory distress. (Ensure that all males and kids 10 and under are asked to expose their chest after consent)
Abdomen	• Gross distension, scars, hernia, swellings may be visualized. Patient/attendant can identify an area of abdominal tenderness.
Neurological	• Higher mental status (alert/drowsy/stupor/coma or MMSE when needed).
	• Neck rigidity via the chin to chest maneuver.^13^
	• Some cranial nerves (e.g. Facial and hypoglossal) can be tested easily by asking patients to follow movements like lifting eyebrows, smiling, tongue protrusion etc.
	• Estimation of power can be gauged e.g. ability to raise limb against gravity.
Skin	• Rashes like herpes zoster, tinea corporis can be seen.
Psychiatry	• Assessment of anxiety and depression via tools like GAD7 and PHQ9 can be done.
Musculoskeletal	• Gait and range of motion of joints can be ascertained after providing specific instructions to patients. Gross joint deformities or swelling may also be visualized.

### Ending the consultation:

- As with any consultation, medication review is important.
- Ensure prescriptions and investigation orders are shared with the patient in a timely fashion i.e. end of the clinic/day and follow up date provided.
- It is useful to write important instructions/advice on prescription

### Situations/Conditions ideal for tele-consults:

- Recent follow-up patients who may not require physical examination.
- Patients with follow-up for non-communicable illnesses like diabetes, hypertension, dyslipidemia, depression and anxiety.
- Older patients who may find access to care difficulty because of decreased mobility

### Problems for which tele-Consult may not be appropriate:

- Conditions that require immediate physical examination like severe abdominal pain, respiratory distress, trauma, acute mental status changes are a few examples

### Teaching in teleclinics

With universities shutdown to maintain student safety, teleclinics provide a safe alternative opportunity for learning. The learning can be in the form of direct observation of doctor-patient interaction or supervised clerking of patients while simultaneously learning to communicate via digital medium. Depending on institutional guidelines, a trainee can either join clinic in person or virtually from a private place to maintain confidentiality.

- During e-consultations it is important to not interrupt a conversation between the trainee and patient, as the communication gets confusing for the patient sitting across the connection.
- The history, diagnosis and plan can be presented with the patient either on mute (after informing them) or the call can be disconnected momentarily (after informing the patient).
- It is best to give feedback to the learner immediately after a tele-consult (like in a usual face to face situation) for things not covered or on critical reasoning skills.

## CONCLUSION

Tele-medicine is likely to become an adjunct strategy for healthcare delivery. A clear process for a tele-consultation is important and specific protocols should be developed by individual organizations based on resources available. As a next step, studies on quality assurance of tele-consultations may help to gauge impact of this new modality in healthcare delivery.

### Authors’ Contribution:

**MA** prepared the manuscript.

**NS** contributed in preparing the manuscript.

**SRS** conceived, reviewed and approved the manuscript.
